# Newcastle disease virus infection in chicken embryonic fibroblasts but not duck embryonic fibroblasts is associated with elevated host innate immune response

**DOI:** 10.1186/s12985-016-0499-1

**Published:** 2016-03-15

**Authors:** Yinfeng Kang, Minsha Feng, Xiaqiong Zhao, Xu Dai, Bin Xiang, Pei Gao, Yulian Li, Yanling Li, Tao Ren

**Affiliations:** College of Veterinary Medicine, South China Agricultural University, 483 Wushan Road, Tianhe District, Guangzhou 510642 P.R. China; Key Laboratory of Animal Vaccine Development, Ministry of Agriculture, Guangzhou, China; Key Laboratory of Zoonosis Prevention and Control of Guangdong, Guangzhou, China; National and Regional Joint Engineering Laboratory for Medicament of Zoonosis Prevention and Control, Guangzhou, China

## Abstract

**Background:**

Chickens and ducks are major hosts of Newcastle disease virus (NDV) with distinct responses to infection. However, whereas ducks are generally asymptomatic or exhibit only mild symptoms following NDV infection and are thus regarded as potential long-term reservoirs of the virus, chickens exhibit severe clinical lesions, transient infections and even death due to NDV infection. These differences may in part result from the host innate immune response to NDV infection.

**Methods:**

To better understand the host innate immune response to NDV infection in avian species, by using the quantitative real-time polymerase chain reaction method we examined the messenger RNA expression levels of immune-related genes in chicken embryonic fibroblasts (CEFs) and duck embryonic fibroblasts (DEFs) when infected with NDV of different pathogenicities.

**Results:**

Gene expression profiles showed that the expression of *IL-1beta*, TNF-α-like factor (*LITAF*) and *interferon (IFN)-beta* was upregulated in both CEFs and DEFs infected with SS-10 and NH-10 viruses or treated with polyinosinic:polycytidylic acid [poly(I:C)], as well as that expression levels were greater in CEFs than in DEFs. The expression of *TLR3*, *TLR7*, *IL-6*, *IFN-alpha*, *IFN-gamma*, *MHC-I* and *MHC-II*, except for *IL-8*, were also greater in CEFs than in DEFs in response to infection to both viruses or treatment with poly(I:C). However, unlike moderate virulent NH-10, highly virulent SS-10 induced greater pattern recognition receptors and cytokines, except for *IFNs*, in CEFs and DEFs.

**Conclusion:**

Results show distinct expression patterns of cytokines, Toll-like receptors and *IFNs* associated with inflammatory immune responses to NDV between species and by virulence.

## Background

Caused by Newcastle disease virus (NDV), Newcastle disease (ND) is one of the most critical diseases in poultry and wild birds, largely due to its high morbidity and mortality, as well as its worldwide distribution and threat of considerable economic losses to avian industries [1]. NDV is a negative-sense, single-stranded and enveloped RNA virus with approximately 15.2 kb genome composed of six genes encoding at least six structural proteins and additional non-structural proteins—namely, hemagglutinin–neuraminidase (HN), nucleoprotein (NP), fusion (F), phosphoprotein (P), matrix (M), RNA-dependent RNA polymerase (L) [2], V, and possibly the W protein, produced by RNA editing of the P coding region [3, 4]. With a wide range of hosts, NDV is known to infect at least 250 bird species through either experimental or natural routes [1]. Given the cleavage site of the F protein and due to the severity of disease, NDV strains in 1-d-old specific pathogen-free (SPF) chickens are categorized as highly virulent (i.e., velogenic), intermediate virulent (i.e., mesogenic) or nonvirulent (i.e., lentogenic) [1, 5] according to the Intracerebral Pathogenicity Index.

Wild waterfowl and shorebirds can act as reservoir hosts, as well as hosts by which viruses with pandemic potential are known to be effectively transmitted to other avian species, and have thus gained attention with the emergence and perpetuation of virulent NDV through serial passage in susceptible animals [6–9]. Even so, few studies have addressed viral pathogenesis and host innate immune response in avian species, thereby leaving gaps in the knowledge of NDV in avian hosts. In particular, chickens and ducks respond to virulent NDV infections differently, and many cases have demonstrated that infection with a specific virulent NDV strain may cause lesions and even death in chickens, whereas a duck infected with the same virus would be asymptomatic and rarely die due to the infection [10, 11]. Moreover, NDV shedding in infected chickens is transient and involves the host’s rapid clearance [12, 13], whereas infected ducks exhibit intermittent, prolonged shedding [11]. Another difference is chickens’ greater probability than ducks of an earlier, stronger humoral immune response to NDV infection [14]. Furthermore, though previous studies have shown that NDV replicates preferentially in both specifies’ respiratory systems and lymphoid tissues, including the lungs, spleen, thymus and bursa of Fabricius [10, 11], only in ducks does NDV’s distribution remain limited to lymphoid tissues [15]. Perhaps more significantly, though having adapted efficient replication in chickens, NDV does not always replicate in ducks, yet depends on its adaptation to different hosts and vice versa. However, to our knowledge, very few studies have compared the viral pathogenesis of or host innate immune responses to the same NDV in chicken and duck embryonic fibroblasts.

At the cellular level, a host’s recognition of viruses is mediated by Toll-like receptors (TLRs), such as TLR3 and TLR7, which recognize viral components and activate intracellular signal transduction pathways. Those processes result in the production of antiviral cytokines such as type I interferons (*IFNs*) and proinflammatory cytokines and chemokine, including *IL-6* and *IL-1beta*, as well as major histocompatibility complexes (MHC) that support host defenses against clearance of viruses [16]. MHCs of classes I and II exhibit an antigen presentation associated with cell-mediated immunity (CMI) that plays an important role in defending T lymphocytes (e.g., cytokine-secreting CD4+ T helper cells and CD8+ cytotoxic T lymphocytes) against viral infection and is essential for viral clearance [17, 18]. Previous studies have reported that in MHC class I and II molecules, pattern recognition receptor (PRRs) and antiviral cytokines were involved in the host innate immune response of avian species, including chickens and ducks, when infected with NDV [19–21]. Nevertheless, very few studies have compared the induction and role of MHC class I and II molecules, PRRs and antiviral cytokines in avian embryo fibroblasts when infected with NDVs of different pathogenicities.

For this study, we selected a model of chicken embryo fibroblasts (CEFs) and duck embryo fibroblasts (DEFs) to observe host innate immune responses in vitro following infection with NDVs of different pathogenicities. To better understand the host immune responses and mechanisms supporting the different pathogeneses of NDV infection of two the highly relevant avian species of chickens and ducks, we compared the expression of cytokines and PRRs, including TLRs and proinflammatory and antiviral cytokines, in response to NDV infection, all with the quantitative real-time polymerase chain reaction (qRT-PCR) method. With that same method, we also examined cell-mediated immune responses and MHC class I and II molecules in CEFs and DEFs.

## Methods

### Ethics statement

This experiment was conducted with the approval of the South China Agricultural University Experimental Animal Welfare Ethics Committee (permit no. 2015–08).

### Cell lines and virus

CEFs and DEFs were obtained from 10-d-old SPF chicken embryos and 11-d-old Pekin duck embryos (South China Agricultural University, Guangzhou, China) as previously described [22]. The CEFs and DEFs were maintained in Dulbecco’s modified Eagle’s medium (DMEM) (Gibco) supplemented with 10 % fetal bovine serum (FBS) (Gibco), 100 U/mL penicillin and 100 ug/mL streptomycin at 37 °C with 5 % CO_2_ until cell density reached approximately 80 % confluence. The two NDV strains used were Duck/CH/GD/SS/10 (SS-10) and Duck/CH/GD/NH/10 (NH-10), genotypes VII and IX, respectively, both duck-adapted viruses characterized well in the Key Laboratory of Animal Disease Control and Prevention of the Ministry of Agriculture, College of Veterinary Medicine [11]. The viruses were inoculated into the allantoic cavity of 10-d-old SPF-embryonated chicken eggs at 37 °C for 3 d according to the standard procedures of the Office International Des Epizooties [1]. Fresh allantoic fluid was collected and stored at −80 °C until used, and virus titers were quantified by plaque assay with both CEFs and DEFs [23].

### Growth characteristics of the two NDVs in CEFs and DEFs

To determine the multicycle growth kinetics of SS-10 and NH-10, CEFs and DEFs in triplicate wells of six-well plates were infected with either virus at a multiplicity of infection (moi) of 1. Following 1 h of adsorption, the cells were washed and covered with DMEM containing 2 % heat-inactivated FBS at 37 °C and 5 % CO_2_. Cell culture supernatant samples were collected and replaced with an equal volume of fresh medium at 6, 12, 24, 36, 48 and 60 h post-infection (p.i.). Virus titers were quantified by a plaque assay on CEFs as previously described [23].

### Virus infection

CEFs and DEFs were seeded 16 h prior to infection in triplicate wells of six-well plates at a cell density of approximately 1.7 × 10^5^ cells/well. The cells were infected with genotype VII SS-10 and genotype IX NH-10, both predominant duck-origin genotypes of NDV strains circulating in Guangdong Province, at an moi of 1 and incubated at 37 °C in a humidified atmosphere containing 5 % CO_2_ for 1 h. Afterward, the growth medium was replaced with DMEM supplemented with 2 % heat-inactivated FBS. Mock-infected cells were regarded as negative controls. CEFs and DEFs were treated with the double-stranded RNA (dsRNA) analog poly(I:C) (Sigma–Aldrich) at a concentration of 20 ug/mL and used as positive controls. At 0, 6, 24 and 36 h p.i., cell monolayers were harvested and stored at −80 °C for RNA extraction.

### RNA extraction and cDNA synthesis

Total RNA was extracted from the infected and negative control embryo fibroblast cells harvested at each time point using the RNeasy Mini RNA Purification Kit (Qiagen, Valencia, CA, USA) according to the manufacturer’s instructions. Viral RNA was extracted from culture supernatants by using an RNA extraction kit (Takara, Japan). RNA in each sample was quantified using an Ultrospec 2000 mass spectrophotometer (Pharmacia Biotech, Uppsala, Sweden). Approximately 2 ug RNA from each sample was treated with DNase to remove genomic DNA and was later reverse-transcribed to cDNA using the SuperScript® III First-Strand Synthesis System (Clontech) according to the manufacturer’s protocol.

### Quantitative real-time polymerase chain reaction (qRT-PCR)

The qRT-PCR method was applied in a final volume of 25 uL using a QuantiFast SYBR Green PCR Master Mix Kit (Qiagen) with specific primers designed with Oligo 7 software (http://www.oligo.net/) based on published sequences [24]. Primers were developed for *IL-1beta*, lipopolysaccharide-induced TNF-α-factor (*LITAF*), *IL-2*, *IL-6*, *IFN* types I and II (*IFN-alpha*, *IFN-beta* and *IFN-gamma*), MHC class I and II molecules, *TLR3*, *TLR7* and *IL-8*, all derived from published sequences. Predicted product sizes are shown in Table 1. All qRT-PCR assays were conducted using the ABI Prism 7500 Fast Real-Time PCR System (Applied Biosystems), which involved predegeneration for 2 min at 50 °C and initial denaturation for 30 s at 95 °C, followed by 40 cycles of 5 s at 94 °C and 34 s at 60 °C, as well as a melt-curve analysis to confirm the specificity of the SYBR green PCR signal. A one-step qRT-PCR assay of viral RNA using NDV P/V/W gene-specific primers was performed as previously described. Cycle threshold (CT) values were converted to viral gene copy numbers following a standard curve generated using cDNA. To rule out genomic contamination, control qRT-PCRs were performed in the absence of reverse transcriptase. Amplified products were run on a gel and extracted using a QIAEX II DNA gel extraction kit (Qiagen, Germany) according to the manufacturer’s instructions. To validate assays, purified products were subcloned into pMD19-T with a TA cloning kit (Clontech, Japan) and sequenced for verification using M13 forward and reverse primers.

**Table 1:** Primer sequences for quantitative real-time polymerase chain reaction

RNA target	Forward primer (5' → 3')	Reverse primer (5' → 3')	Product size (bp)	GenBank accession no.
Chicken				
GAPDH	CCTCTCTGGCAAAGTCCAAG	CATCTGCCCATTTGATGTTG	200	NM_204305
TLR3	ACAATGGCAGATTGTAGTCACCT	GCACAATCCTGGTTTCAGTTTAG	123	NM_001011691
TLR7	TGTGATGTGGAAGCCTTTGA	ATTATCTTTGGGCCCCAGTC	218	DQ780342
IL-1β	GCTCTACATGTCGTGTGTGATGAG	TGTCGATGTCCCGCATGA	80	NM204524
IL-6	CCTGTTCGCCTTTCAGACCT	GGGATGACCACTTCATCGGG	171	EU170468
IL-8	ATTCAAGATGTGAAGCTGAC	AGGATCTGCAATTAACATGAGG	196	DQ393272
LITAF	CCGCCCAGTTCAGATGAGTT	GCAACAACCAGCTATGCACC	130	AY765397
IFN-a	ATGCCACCTTCTCTCACGAC	AGGCGCTGTAATCGTTGTCT	387	EU367971
IFN-r	TGAGCCAGATTGTTTCGATG	CTTGGCCAGGTCCATGATA	248	DQ906156
MHC-I	AAGAAGGGGAAGGGCTACAA	AAGCAGTGCAGGCAAAGAAT	222	NM001031338
MHC-II	CTCGAGGTCATGATCAGCAA	TGTAAACGTCTCCCCTTTGG	312	DQ008588
Duck				
GAPDH	ATGTTCGTGATGGGTGTGAA	CTGTCTTCGTGTGTGGCTGT	176	AY436595
TLR3	GAGTTTCACACAGGATGTTTAC	GTGAGATTTGTTCCTTGCAG	200	NM_001310782
TLR7	CCTTTCCCAGAGAGCATTCA	TCAAGAAATATCAAGATAATCACATCA	154	AY940195
IL-1β	TCGACATCAACCAGAAGTGC	GAGCTTGTAGCCCTTGATGC	185	DQ393268
IL-6	TTCGACGAGGAGAAATGCTT	CCTTATCGTCGTTGCCAGAT	150	AB191038
IL-8	AAGTTCATCCACCCTAAATC	GCATCAGAATTGAGCTGAGC	174	AB236334
LITAF	ACAGGACAGCCTATGCCAAC	CATCTGAACTGGGCGGTCAT	96	EU375296
IFN-a	TCCTCCAACACCTCTTCGAC	GGGCTGTAGGTGTGGTTCTG	232	EF053034
IFN-r	GCTGATGGCAATCCTGTTTT	GGATTTTCAAGCCAGTCAGC	247	AJ012254
MHC-I	GAAGGAAGAGACTTCATTGCCTTGG	CTCTCCTCTCCAGTACGTCCTTCC	196	AB115246
MHC-II	CCACCTTTACCAGCTTCGAG	CCGTTCTTCATCCAGGTGAT	229	AY905539
Chicken and Duck IFN-β	CCTCAACCAGATCCAGCATT	GGATGAGGCTGTGAGAGGAG	259	AY831397

### Data and statistical analysis

The cDNA sample of each CEF and DEF was tested in triplicate. Relative expression levels were calculated according to the 2^-△△CT^ method, which involved using glyceraldehyde-3-phosphate-dehydrogenase (GAPDH) as the endogenous control to normalize the level of target gene expression [25]. Data were expressed as *M* ± *SD*. Growth characteristics of each group were analyzed using an unpaired Student’s *t*-test, and differences between the means of the CEF and DEF target genes were evaluated using two-way analysis of variance (ANOVA) followed by Duncan’s test. All *p* values less than .05 were considered to be statistically significant. Statistical analyses for *M*, two-way ANOVA and *SD* were conducted by using Prism 6 (GraphPad Software, Inc., San Diego, CA, USA).

## Results

### Replication kinetics of CEFs and DEFs infect with SS-10 and NH-10

**Fig. 1:**
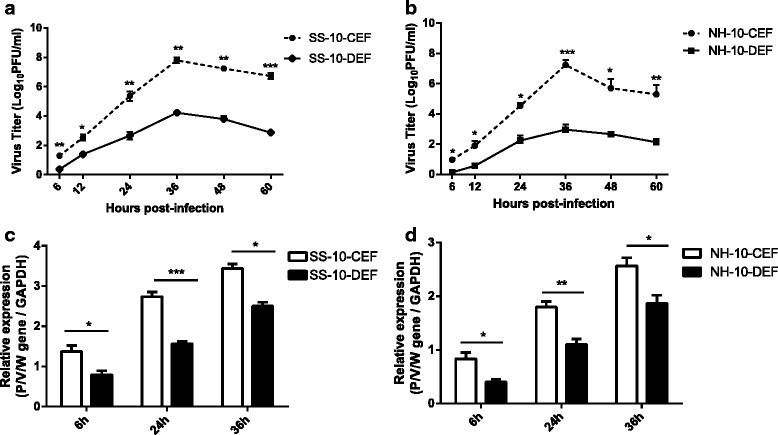
Comparison of multicycle growth kinetics and progeny virus outputs of SS-10 and NH-10 strains on chicken embryo fibroblasts (CEFs) and duck embryo fibroblasts (DEFs). CEFs and DEFs were inoculated with SS-10 (**a**) and NH-10 (**b**) at a moi of 1, and cell supernatant was sampled at the time points indicated. Virus titers in the cell supernatant were determined in CEFs via plaque assay. SS-10 (**c**) and NH-10 (**d**) at a moi of 1 conferred higher levels of accumulation of P/V/W gene RNA in CEFs than in DEFs. Values represent averages of the results from three independent experiments with standard error bars. *p < .05, **p < .01, ***p < .001, as determined by an unpaired Student’s *t*-test

The multicycle growth kinetics and replication magnitude of SS-10 and NH-10 were determined in CEFs and DEFs by using a plaque assay. CEFs and DEFs were inoculated with each virus at a moi of 1, and cell supernatants were harvested at the time points indicated. As shown in Fig. 1a and b, SS-10 replicated more efficiently and had a significantly higher titer in CEFs and DEFs than NH-10 at each time point, though both viruses achieved similar maximum titers at 36 h p.i. On the whole, the virus titers of both strains were higher in CEFs than in DEFs during the 60 h of testing (Fig. 1a and b). Additionally, CEFs and DEFs were infected with SS-10 and NH-10 at a moi of 1 over a period of 36 h. Normalized to the endogenous control, viral P/V/W gene RNA accumulation in DEFs was consistently less than that in the corresponding CEFs for two NDV isolates (Fig. 1c and d).

### Expression of TLR genes in NDV-infected CEFs and DEFs

**Fig. 2:**
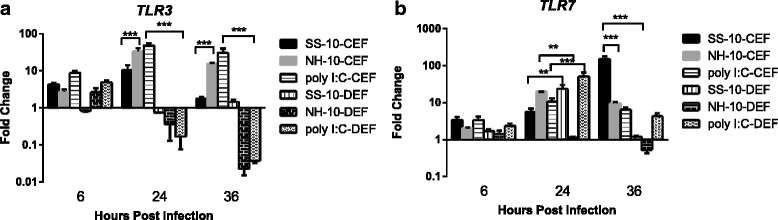
The expression of TLRs *TLR3* (**a**) and *TLR7* (**b**) in chicken embryo fibroblasts (CEFs) and duck embryo fibroblasts (DEFs) following infection with SS-10 and NH-10 or treatment with poly(I:C) at 6, 24 and 36 h p.i. Data represent the mean fold change expression of either CEFs and DEFs compared with mock-infected controls after normalization to the expression of the housekeeping gene glyceraldehyde-3-phosphate-dehydrogenase. Significance was analyzed with two-way analyses of variance between the SS-10 and NH-10 groups in CEFs and DEFs at each time point (*p < .05, *p < .01, *p < .001). Error bars represent *SD*

We compared the expression levels of TLR genes *TLR3* and *TLR7* in CEFs and DEFs infected with SS-10 and NH-10 at 6, 24 and 36 h p.i. Compared to those in mock-infected samples, the expression levels of *TLR3* and *TLR7* in CEFs were upregulated at 6 h p.i. and peaked at 24 h p.i., with the exception of *TLR7*’s expression level induced by SS-10 at 36 h p.i. Thereafter, the levels decreased slightly at 36 h p.i. when either infected with both viruses or treated with poly(I:C), as shown in Fig. 2a and b. In DEFs, the expression levels of *TLR3* and *TLR7* induced by both viruses and positive control poly(I:C) clearly exhibited patterns of expression. For *TLR3*, the expression level was downregulated at 6 and 24 h p.i. (0.85- and 0.75-fold, respectively) and upregulated at 36 h p.i. (1.46-fold) in response to SS-10 infection, whereas the expression level of *TLR3* was upregulated at 6 h p.i. and downregulated at 24 and 36 h p.i. following infection with NH-10 or after stimulation with poly(I:C) (Fig. 2a). Meanwhile, the expression level of *TLR7* was upregulated throughout the period of infection, except at 36 h p.i. induced by NH-10, in response to infection with both viruses or treatment with poly(I:C) (Fig. 2b). Notably, the expression levels of TLR genes *TLR3* and *TLR7* in CEFs induced by both viruses and poly(I:C) were greater than in DEFs throughout the testing period, whereas *TLR7* was slightly increased in DEFs at 24 h p.i. following stimulation with dsRNA analogs (poly(I:C)).

### Differential expression of proinflammatory cytokines and chemokines in CEFs and DEFs infected with NDV of different pathogenicities

**Fig. 3:**
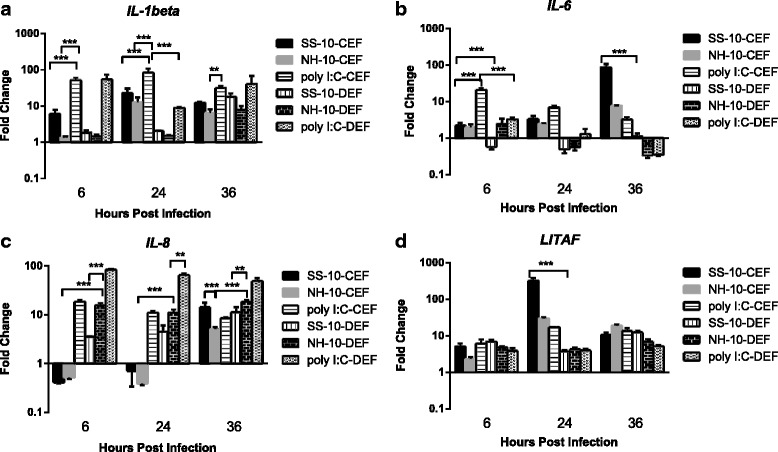
Proinflammatory cytokines *IL-1beta* (**a**), *IL-6* (**b**), *LITAF* (**c**) and chemokine *IL-8* (**d**) expression in chicken embryo fibroblasts (CEFs) and duck embryo fibroblasts (DEFs) following infection with SS-10 and NH-10 or treatment with poly(I:C) at 6, 24 and 36 h p.i. Data represent the mean fold change expression of either CEFs and DEFs compared with mock-infected controls after normalization to the expression of the housekeeping gene glyceraldehyde-3-phosphate-dehydrogenase. Significance was analyzed with two-way analyses of variance between the SS-10 and NH-10 groups in CEFs and DEFs at each time point (*p < .05, **p < .01, ***p < .001). Error bars represent *SD*

To compare proinflammatory and Th1-associated cytokines and chemokines in CEFs and DEFs infected by SS-10 and NH-10, cytokines and chemokines such as *IL-1beta*, *IL-6*, *IL-8* and *LITAF* were measured early during either NDV infection or treatment with poly(I:C). Compared to uninfected cells, CEFs and DEFs exhibited upregulated expression levels of *IL-1beta* and *LITAF* in response to infection with SS-10 and NH-10 or treatment with poly(I:C) during the testing period (Fig. 3a and d), though the expression levels of *IL-1beta* and *LITAF* showed different expression patterns at each time point for both infected CEFs and DEFs (Fig. 3a and d). In CEFs, the expression of both *IL-1beta* and *LITAF* was upregulated when induced by infection with SS-10 and NH-10 at all time points and peaked at 24 h p.i. (22.87- and 12.66-fold versus 319.48- and 21.13-fold, respectively), but was greater for SS-10 than NH-10. Albeit also upregulated, *IL-1beta* expression in DEFs was induced at a slightly weaker level and peaked at 36 h p.i. (17.93- and 7.88-fold, respectively), induced by both viruses. *LITAF* expression in DEFs was also induced 6.93- and 4.84-fold at 6 h p.i., respectively, decreased slightly at 24 h p.i. (3.71- and 4.25-fold, respectively) and peaked at 36 h p.i. (12.33- and 6.86-fold, respectively), when infected with SS-10 and NH-10, yet was induced at a far lower level than with CEFs during the observation period (Fig. 3d). The expression level of IL-6 was upregulated in CEFs during the period of infection, peaking at 36 h p.i. and 8 h p.i. (88.22-, 7.60- and 21.21-fold, respectively) in response to infection with SS-10 and NH-10 or treatment with poly(I:C) compared to uninfected CEFs. However, it was downregulated at 6 and 24 h p.i. and maintained baseline level at 36 h p.i. in DEFs induced by SS-10. It was moreover upregulated 2.49-fold at 6 h p.i. and downregulated between 24 and 36 h p.i. (0.56- and 0.34-fold, respectively) in DEFs induced by NH-10 (Fig. 3b). Remarkably, SS-10 induced the expression of proinflammatory cytokine *IL-6* to a greater extent than NH-10 during the testing period, whereas it was not statistically significant in DEFs (Fig. 3b).

The expression level of chemokine *IL-8* was suppressed at 6 and 24 h p.i., yet upregulated at 36 h p.i. in CEFs when induced by SS-10 and NH-10. However, the expression level of *IL-8* showed a different pattern of increase at 6 h p.i. in DEFs and maintained the same tendency at 24 and 36 h p.i. when infected with SS-10 and NH-10, but was higher than that of CEFs at each time point. By contrast, the expression level of *IL-8* was upregulated when treated with poly(I:C) in CEFs and DEFs during the testing period, but was higher in DEFs than in CEFs (Fig. 3c).

In sum, these results indicate that the expression levels of proinflammatory cytokines and chemokines *IL-1beta*, *IL-6*, *IL-8* and *LITAF* in CEFs and DEFs showed different patterns following infection with NDVs of different pathogenicities.

### Differential expression of antiviral cytokines in CEFs and DEFs in NDV-infected embryo fibroblast cells

**Fig. 4:**
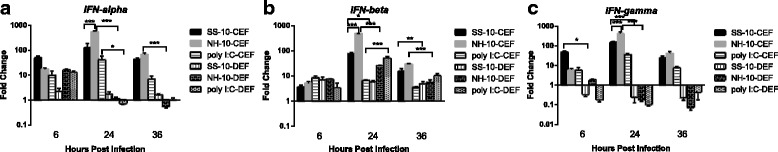
Type I and II *interferon* (*IFN*)*-alpha* (**a**), *IFN-beta* (**b**) and *IFN-gamma* (**c**) expression in chicken embryo fibroblasts (CEFs) and duck embryo fibroblasts (DEFs) following infection with SS-10 and NH-10 or treatment with poly(I:C) at 6, 24 and 36 h p.i. Data represent the mean fold change expression of either CEFs and DEFs compared with mock-infected controls after normalization to the expression of the housekeeping gene glyceraldehyde-3-phosphate-dehydrogenase. Significance was analyzed with two-way analyses of variance between the SS-10 and NH-10 groups in CEFs and DEFs at each time point (*p < .05, **p < .01, ***p < .001). Error bars represent *SD*

As is well known, the IFN system is the most important host defense mechanism during infection with viral pathogens, for it controls and surpasses viral replication and modulates innate immune responses. In that context, in this study we compared the induction of IFN type I and II (*IFN-alpha*, *IFN-beta* and *IFN-gamma*) responses in CEFs and DEFs triggered by the SS-10 and NH-10 of different pathogenicities at the early stage of infection and that exhibited a similar species-dependent immune response. *IFN-alpha*, *IFN-beta* and *IFN-gamma* expression was upregulated in CEFs throughout the experiment period and peaked at 24 h p.i. (126.96- and 535.49-fold, 77.32- and 474.78-fold and 152.19- and 409.92-fold, respectively) induced by SS-10 and NH-10 when compared to mock-infected controls, yet was greater for NH-10 than SS-10 (Fig. 4a, b and c). In DEFs, the expression level of *IFN-alpha* was upregulated at 6 h p.i. (2.21- and 16.36-fold, respectively), gradually decreased to baseline level at 24 h p.i. (1.72- and 1.14-fold, respectively) and decreased further at 36 h p.i. (1.62- and 0.55-fold, respectively) after infection with SS-10 and NH-10 (Fig. 4a). The expression level of *IFN-beta* was upregulated during the tested period and peaked at both 6 and 24 h p.i. (7.09- and 25.28-fold, respectively) induced by both viruses (Fig. 4b). The expression level of *IFN-gamma* was downregulated for the duration of the study induced by SS-10; however, it was upregulated at 6 h p.i. (1.83-fold) and then downregulated at the other time points induced by NH-10 (Fig. 4c). In brief, these results demonstrate that the induction of rapid and robust type I and II IFNs in CEFs is far higher than in DEFs following challenge with virulent NDV and is lower for SS-10 than NH-10.

### MHC class I and II molecule expression preference by embryo fibroblast cells

**Fig. 5:**
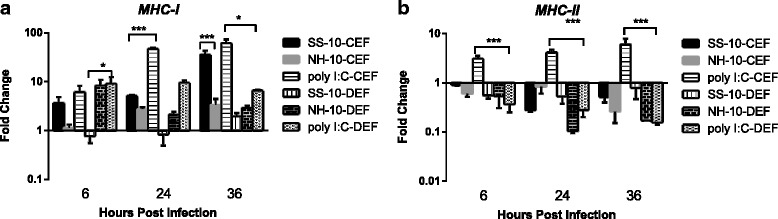
*MHC I* (**a**) and *II* (**b**) expression in chicken embryo fibroblasts (CEFs) and duck embryo fibroblasts (DEFs) following infection with SS-10 and NH-10 or treatment with poly(I:C) at 6, 24 and 36 h p.i. Data represent the mean fold change expression of either CEFs and DEFs compared with mock-infected control after normalization to the expression of the housekeeping gene glyceraldehyde-3-phosphate-dehydrogenase. Significance was analyzed with two-way analyses of variance between the SS-10 and NH-10 groups in CEFs and DEFs at each time point (*p < .05, **p < .01, ***p < .001). Error bars represent *SD*

To compare the expression of MHC class I and II molecules, we examined their relative expression in CEFs and DEFs by qRT-PCR during early-phase NDV infection. MHC class I molecule expression was upregulated in CEFs and DEFs induced by SS-10 and NH-10 or treatment with poly(I:C) during the period of infection, except at 6 and 24 h p.i. in DEFs in response to infection with SS-10 (Fig. 5a). Importantly, the expression level of MHC class I induced by SS-10 was higher than NH-10 in CEFs, yet lower for SS-10 than NH-10 in DEFs at all time points (Fig. 5a). The expression level of MHC class II molecules was downregulated when induced by SS-10 and NH-10 or following stimulation with poly(I:C) for the duration of the study, whereas it was upregulated in CEFs at all time points when treated with poly(I:C) compared to mock-infected controls (Fig. 5b). Most importantly, MHC class I and II molecule expression in CEFs and DEFs showed different expression patterns that were associated with different pathogenicities against ND.

## Discussion

ND is a highly devastating viral disease in avian species that results high mortality and morbidity [1]. A wide variety of birds infected with NDV have been reported, though different species have exhibited different pathogenicities following infection with specific NDVs [26, 27]. Moreover, various NDV strains induce different host innate immune responses in specific animals [20, 21]. In this study, according to the results of replication kinetics in CEFs and DEFs when infected by virulent NDVs, the titers in CEFs were higher than in DEFs at each time point. However, the reason for this varying replication ability between the two species remains unknown, as does the role that the difference of disease severity plays in host pathogen immune responses to NDV infection.

Studies have demonstrated that NDV infection in immune cells—for instance, peripheral blood mononuclear cells and macrophages—results in extremely robust proinflammatory and antiviral cytokine induction both in vivo and in vitro [14, 20, 28, 29]. The expression of cytokines such as *LITAF*, *IL-1beta*, *IL-8* and *IL-6* in splenic leukocytes, macrophages and lymphoid tissues in chickens, ducks, geese and pigeons immediately are distinct in response to NDV infection [19–21, 30]. Our results show that proinflammatory cytokines *IL-1beta*, *IL-6*, chemokine *IL-8*, antiviral cytokines *IFNs* and PRRs such as *TLR3* and *TLR7*, as well as MHC class I and II molecules, show different expression patterns, whereas *LITAF* is indistinct between the two species. The production of higher inflammatory immune responses to CEFs furthermore contrasts that of DEFs, which might at least partially explain the high morbidity and mortality of these birds following virulent NDV infection. Positive control stimulation with poly(I:C) in embryo fibroblast cultures also shows that differences in species are specific to the NDV. The increased production of proinflammatory cytokines and the severity of the cytopathic effect in CEFs when compared with DEFs following NDV infection might provide a plausible explanation for retinoic acid–inducible gene I (RIG-I) absence in CEFs, a viral RNA sensor that plays a crucial role in IFN-mediated antiviral immunity responses [31].

Our study has moreover shown an elevated induction of type I and II IFNs in CEFs and a weak production of type I and II IFNs in DEFs in response to NDV infection, which suggests the relative susceptibility of CEFs to NDV infection over DEFs, as consistent with previous observations of pathogenicity variation in different birds [11, 27]. The infection of CEFs and DEFs with SS-10 resulted in the weak induction of type I IFN compared to NH-10, likely due to cysteine-rich C terminus deletion in its V protein, which is critical for blocking IFN induction in embryo fibroblast cells [32]. The interaction of V and laboratory of genetics and physiology 2 or melanoma differentiation-associated gene 5 required for targeting STAT1 for degradation results in the inhibition of IFN signaling in chicken cells and Vero cells [32–34]. Rue et al. have shown that highly virulent NDV induces higher host innate immune responses compared with avirulent NDV in chicken spleens [19]. In our study, we found that CEFs induce significantly higher levels of IFN than DEFs following virus infection when compared with the expression levels of type I and II IFNs (Fig. 4). Studies have shown that the infection of chickens with virulent NDV resulted in a weak induction of IFNs that correlated with a longer shedding period, higher virus titers and greater disease severity [11, 35]. According to the above results, we speculate that the higher overall induction of IFNs by CEFs following infection with virulent NDV reflects what happens at the level of the organism, meaning shorter shedding and more rapid viral clearance in chickens and and lower virus replication and weaker viral clearance in ducks, as well as a longer shedding period.

We also found that the *IL-6* mRNA transcript was upregulated in CEFs with both viruses and in treatment with polyI:C. By contrast, it was downregulated in DEFs with virulent NDV infection at 24 h p.i. (Fig. 3b). *IL-6* mediates the limit and containment of NDV replication in the spleen of infected chickens during the early phase of infection, namely through the activation of host innate immune mechanisms such as macrophages and TLRs, which can contribute to pathological damage observed in NDV-infected chickens [19, 36]. Studies have shown that *TLR3* plays a fundamental role in the expression of proinflammatory cytokines such as *IL-6* and *IL-1beta* in fibroblasts or classical dendritic cells derived from *TLR3*-deficient mice after infection with NDV [37]. *TLR3*-deficient mice exhibited prolonged survival accompanied with reduced proinflammatory cytokines *IL-6* and *IFNs* when infected with the Sendai virus, an enveloped animal virus of the family *Paramyxoviridae* similar to NDV [37]. Based on our results, there is a positive correlation in CEFs and DEFs infected with NDV in terms of the expression level of *IL-6* and *TLR3*, which suggests the fundamental role of *IL-6* in NDV pathogenesis.

TLRs such as *TLR3* and *TLR7* play an essential role in producing inflammatory cytokines and IFNs, as well as in activating host innate immune responses by triggering pathogen-associated molecular patterns, including the nucleic acid of RNA viruses such as NDV in mammals, insects and domestic poultry [38]. Yilmaz et al. [39] have reported that chicken *TLR3* and *TLR7* were highly expressed in the kidneys, liver, heart, spleen, intestines, lungs and oviduct, whereas *TLR3* mRNA in ducks was only highly expressed in the spleen and lungs, moderately expressed in the intestines, liver and kidneys, poorly expressed in the heart, brain, bursa, and skin and not expressed whatsoever in muscle tissue [40]. Duck *TLR7* mRNA was moreover highly expressed in the spleen, lungs and bursa, poorly expressed in the kidneys and liver, and not expressed whatsoever in the heart and brain, which is distinct from the expression patterns of *TLR3* and *TLR7* in chickens [41]. Our results reveal distinct expression patterns for *TLR3* and *TLR7* in CEFs and DEFs when exposed to NDV infection or treatment with polyI:C (Fig. 2a and b). The observed difference in *TLR3* and *TLR7* expression may be due to differences in the genome of tissues of chickens and ducks, or else the presence of resident cells that express *TLR3* and *TLR7* receptors absent in chickens.

## Conclusions

In sum, our results reveal differences in the mRNA expression levels of TLRs, proinflammatory cytokines, *IFNs* and other immune-related genes between CEFs and DEFs in response to infection with two NDVs with different virulence or treatment with poly(I:C). Our findings also highlight that differential modulation of the host response by NDV strains of different virulence could be an important aspect of NDV pathogenesis. However, to further evaluate virus-specific differences in avian species, more comparative studies need to assess differences in host innate immune responses in avian species following NDV infection.
